# A Functionally Significant Polymorphism in *ID3* Is Associated with Human Coronary Pathology

**DOI:** 10.1371/journal.pone.0090222

**Published:** 2014-03-06

**Authors:** Ani Manichaikul, Stephen S. Rich, Heather Perry, Joseph Yeboah, Michelle Law, Molly Davis, Matthew Parker, Michael Ragosta, Jessica J. Connelly, Coleen A. McNamara, Angela M. Taylor

**Affiliations:** 1 Center for Public Health Genomics, University of Virginia, Charlottesville, Virginia, United States of America; 2 Division of Biostatistics and Epidemiology, Department of Public Health Sciences, University of Virginia, Charlottesville, Virginia, United States of America; 3 Cardiovascular Research Center, University of Virginia, Charlottesville, Virginia, United States of America; 4 Department of Internal Medicine/Cardiology, Wake Forest University Health Sciences, Winston Salem, North Carolina, United States of America; 5 Division of Cardiovascular Medicine, Department of Medicine, University of Virginia, Charlottesville, Virginia, United States of America; Virginia Commonwealth University, United States of America

## Abstract

**Aims:**

We previously identified association between the *ID3* SNP rs11574 and carotid intima-media thickness in the Diabetes Heart Study, a predominantly White diabetic population. The nonsynonymous SNP rs11574 results in an amino acid substitution in the C-terminal region of ID3, attenuating the dominant negative function of ID3 as an inhibitor of basic HLH factor E12-mediated transcription. In the current investigation, we characterize the association between the functionally significant polymorphism in *ID3*, rs11574, with human coronary pathology.

**Methods and Results:**

The Multi-Ethnic Study of Atherosclerosis (MESA) is a longitudinal study of subclinical cardiovascular disease, including non-Hispanic White (n = 2,588), African American (n = 2,560) and Hispanic (n = 2,130) participants with data on coronary artery calcium (CAC). The Coronary Assessment in Virginia cohort (CAVA) included 71 patients aged 30–80 years, undergoing a medically necessary cardiac catheterization and intravascular ultrasound (IVUS) at the University of Virginia. *ID3* SNP rs11574 risk allele was associated with the presence of CAC in MESA Whites (*P* = 0.017). In addition, the risk allele was associated with greater atheroma burden and stenosis in the CAVA cohort (*P* = 0.003, *P* = 0.04 respectively). The risk allele remained predictive of atheroma burden in multivariate analysis (Model 1: covariates age, gender, and LDL, regression coefficient = 9.578, SE = 3.657, p = 0.0110; Model 2: covariates Model 1, presence of hypertension, presence of diabetes, regression coefficient = 8.389, SE = 4.788, p = 0.0163).

**Conclusions:**

We present additional cohorts that demonstrate association of *ID3* SNP rs11574 directly with human coronary artery pathology as measured by CAC and IVUS: one a multiethnic, relatively healthy population with low levels of diabetes and the second a predominantly White population with a higher incidence of T2DM referred for cardiac catheterization.

## Introduction

Coronary artery disease remains the leading cause of death in the United States and most developed countries. Despite rapid advances in therapies aimed at decreasing atherosclerotic disease, significant cardiovascular risk remains. Thus, it is critical to translate novel murine findings into humans with the ultimate goal of creating novel therapies that further reduce cardiovascular risk. To do so requires careful phenotyping of human atherosclerosis at the level of coronary artery pathology. Intravascular ultrasound (IVUS), an invasive imaging study, has become the gold standard for defining the extent of human coronary atherosclerotic burden. While quantitative coronary angiography detects only late disease as evidenced by luminal stenoses, IVUS can directly and precisely quantify disease burden within the artery wall even prior to luminal compromise or presence of calcification [1]. Further, disease burden as measured by IVUS is predictive of cardiovascular events and has also emerged as a strong surrogate marker for events [2], [3]. Coronary artery calcium (CAC) as measured by computed tomography (CT) has emerged as a strong noninvasive surrogate marker for disease burden and event risk. Indeed, in intermediate risk populations, CAC has greater predictive ability than other noninvasive predictors of cardiovascular risk [4].

Research over the last decade has enjoyed major breakthroughs in our understanding of atherosclerotic cardiovascular disease [5]–[8]. Yet, most of these novel insights have come from studies in genetically modified mouse models and corroboration of such pathways in human atherosclerotic disease has been lacking [9]. Sequencing of the human genome and the explosion of the field of human genetics provides opportunities to explore genes implicated in atherosclerosis pathogenesis in murine models in human studies. Statistical associations of single nucleotide polymorphisms (SNPs) with disease provide support that these genes may be involved in human disease pathogenesis [10]. Yet, most SNPs are located in non-coding regions making it difficult to study the relationship between the SNP, the functional impact of the SNP, and disease pathogenesis.

The dominant negative helix-loop-helix (HLH) transcription regulator, ID3, has emerged as an important upstream regulator of atheroprotective pathways in immune and vessel wall cells [11]–[13]. Identification of a SNP in the coding region of the human *ID3* gene, associated with subclinical atherosclerosis in the Diabetes Heart study [11], raises the interesting possibility that *ID3* may have a role in human atherosclerotic disease. This nonsynonymous SNP results in a marked attenuation in the ability for ID3 to affect downstream transcription [11]. Importantly, the allele frequency of the SNP is as high as 48% in White diabetic populations, 28% in HapMap Whites (CEU) and 30% in HapMap Mexican (MEX) populations. ID3 and the bHLH factors it regulates have been implicated in the regulation of gene pathways that mediate cellular growth, differentiation, survival and inflammatory gene expression, supporting the notion that a functional SNP in this upstream pathway regulator could impact on a complex polygenic process such as atherogenesis.

We previously identified an association between the *ID3* SNP rs11574 and carotid intima-media thickness (cIMT) in a predominantly White diabetic population. Here we present two additional cohorts that demonstrate the association of the SNP directly with human coronary artery pathology as measured by CAC and IVUS: the first a multiethnic, relatively healthy population with low levels of diabetes and the second a predominantly White population with a higher incidence of T2DM referred for cardiac catheterization.

## Methods

### Ethics statement

All MESA participants gave written informed consent, including consent to participate in genetic studies. The MESA study was conducted under Institutional Review Board approval at all study sites, including the Cedars-Sinai Medical Center and the University of Virginia. Written informed consent was obtained for all participants of the CAVA study, which was performed under protocol approved by the University of Virginia Institutional Review Board. All methodology was compliant with the principles set forth in the Declaration of Helsinki.

### MESA Study Design

The Multi-Ethnic Study of Atherosclerosis (MESA) is a longitudinal study of subclinical cardiovascular disease that seeks to determine which risk factors predict progression of subclinical disease or progression to clinically overt cardiovascular disease [14]. 6,814 participants free of clinically defined cardiovascular disease were recruited from six field centers across the United States from 2000–2002. Participants were categorized into race/ethnic groups by self-report, and 38% of the recruited participants are non-Hispanic Whites, 28% African-American, 22% Hispanic, and 12% Asian, predominantly of Chinese descent.

Participants recruited by the MESA cohort (6,814) and two ancillary studies – MESA Family Study (MESAFS with 2,128 participants from 528 families) and MESA Air (5,479 participants from MESA, 257 external to MESA, and 490 from MESAFS) were genotyped in 2009 using the Affymetrix Human SNP array 6.0 (∼1 million SNPs). Genotyping and quality control are described in greater detail in the **Supplementary Methods** in **File S1**.

### CAVA Study Design

The Coronary Assessment in Virginia cohort (CAVA) included 71 patients between the ages of 30 and 80 undergoing a medically necessary cardiac catheterization. Patients were excluded if they had any of the following: a current acute coronary syndrome, cancer of any type, autoimmune disease of any type or on immunosuppressive therapy, prior organ transplantation, anemia, pregnancy, HIV infection, or no vessel suitable for IVUS. All protocols and procedures were approved by the Institutional Review Board at the University of Virginia (IRB HSR #14620).

### Phenotyping of MESA and CAVA participants

The MESA cohort measures of subclinical atherosclerosis (SCA) included the presence or absence of CAC, as a dichotomous phenotype. Participants were also assessed for risk factors of subclinical atheroslcerosis, diabetes status, and hypertension.

For CAVA participants, intravascular ultrasound in accordance with the standards of the American College of Cardiology for image acquisition on a non-infarct related artery [15]. The first 40 mm of the artery were used for analysis. The LAD was preferentially analyzed unless there was a contraindication such as tortuosity. Plaque and cross-sectional area were performed at each millimeter and atheroma burden was calculated per segment. Quantitative coronary angiography was also performed for each of the CAVA participants, and percent stenosis was determined using automated edge-detection software by a blinded reader.

Details regarding the phenotyping for MESA and CAVA participants are included in the **Supplementary Methods** in **File S1**.

### Statistical analyses in MESA and CAVA

For MESA, the SNP rs11574 was imputed based on the genome-wide genotype data, and statistical association was assess for rs11574 with CAC, after adjustment for age, sex, study site and principal components of ancestry. For IVUS, rs11574 was genotyped directly and statistical analysis was performed with adjustment for age, gender, LDL, hypertension, and diabetes. Details for the statistical analyses are presented in the **Supplementary Methods** in **File S1**.

## Results

### Characteristics of the study samples

Baseline characteristics and atherosclerosis risk factors for MESA Whites and CAVA participants are summarized separately by *ID3* SNP rs11574 genotype in **Table 1**. Similar measures are also shown for MESA African American and Hispanic American participants in **Table S1** in **File S1**. While the MESA Whites and CAVA are comparable in terms of gender and age distributions, the MESA participants are relatively healthier with notably lower BMI (median 26.9 for ID3 rs11574 risk allele carriers) compared to CAVA (median 32.1 for carriers). Prevalence of diabetes and hypertension are also lower in MESA compared to CAVA. MESA participants have higher LDL levels than CAVA, reflecting the fact that only about 20% of MESA participants are on lipid lowering medication, compared to 77% of CAVA participants on statins. Among MESA participants, the prevalence of CAC was highest in Whites (58.9% of risk allele carriers, **Table S1 in File S1**) compared to African Americans (49.4% of risk allele carriers) and Hispanics (46.3% of risk allele carriers).

**Table 1:** Characteristics of MESA Whites and IVUS participants, by rs11574 genotype.

	Cohort			
	MESA Whites		CAVA	
	Ancestral allele	Risk allele carriers	Ancestral Allele	Risk allele carriers
**Participant characteristics** *				
No. subjects	1517	1071	48	22
Women	770 (50.8)	566 (52.8)	23 (47.9)	15 (68.1)
Age, years	63.0 [54.0, 71.0]	63.0 [54.0, 71.0]	60.5 [51.5,66.5]	63.0 [54.0,70.0]
BMI, kg/m^2^	27.2 [24.3, 30.6]	26.9 [24.1, 30.3]	31.3 [28.3,39.3]	29.5 [26.8,37.9]
LDL-C, mg/dL	115.0 [95.0, 135.0]	116.0 [96.0, 137.0]	87.0 [68.5,117.5]	80.5 [64.0,103.0]
HDL-C, mg/dL	49.0 [40.0, 61.0]	50.0 [42.0, 61.0]	36.0 [29.5, 45]	36.0 [27, 43]
Triglycerides, mg/dL	114.0 [77.0, 163.0]	112.0 [76.0, 163.0]	114.5 [65.5, 186.0]	103.0 [76.0, 152.0]
Diabetes, n (%)	105 (6.9)	59 (5.5)	31 (64.6)	18 (81.8)
Hypertension, n (%)	582 (38.4)	412 (38.5)	37 (77.1)	17 (77.2)
Lipid medication, n (%)†	305 (20.1)	184 (17.2)	37 (77.1)	17 (77.2)

Data are presented as n (%) for binary measures or median [IQR] for continuous measure. “Ancestral allele” and “Risk allele carriers” refer to rs11574 genotypes. “Ancestral allele” denotes homozygote for the common allele (CC) and risk allele carrier indicates heterozygote (CT) or homozygote for the less frequent allele (TT). For MESA, rs11574 was imputed, so we use genotype dosage (i.e. the estimated number of copies of the risk allele) to classify individuals as ancestral allele (dosage in [0, 0.5)) vs. risk allele carriers (dosage in [0.5, 2]).

*Summary statistics are reported for the subset of individuals with data available for at least one of the subclinical atherosclerosis phenotypes.

†In MESA, data are available for “any lipid lowering medication”. For CAVA, these data represent statin use.

### *ID3* SNP rs11574 and CAC in MESA

Our previous work indicating association of cIMT with rs11574 was completed in the Diabetes Heart Study, an entirely White cohort. Therefore, we designated analysis of MESA Whites as our primary analysis, and further examined association in other race/ethnic groups from MESA in secondary analyses. We performed genetic association analysis of the *ID3* SNP rs11574 with measures of SCA in MESA with race/ethnic stratified analyses in White, African American and Hispanic participants, as well as a combined analysis across race/ethnic groups by meta-analysis. We observed statistically significant association of rs11574 with CAC in MESA Whites using a basic regression model that included covariates age, gender, study site, and PCs of ancestry (**Table 2**, *P* = 0.017). This effect was maintained in an extended regression model that added LDL as a covariate (*P* = 0.012). Although we did not observe statistically significant association of rs11574 with CAC in MESA African American or Hispanic cohorts, the direction of effect was the same across all three ethnic groups, such that we observed statistically significant association of *ID3* rs11574 with CAC in meta-analysis across ethnic groups (*P* = 0.017 under the basic regression model, *P* = 0.016 after further adjustment for LDL).

**Table 2:** Detailed race/ethnic-specific results for genetic association of rs11574 with coronary artery calcium (CAC) in MESA.

		Basic Model				Basic Model + LDL			
Group	Allele freq.	N	Beta	SE	P-value	N	Beta	SE	P-value
White	0.239	2468	0.247	0.104	0.017	2432	0.263	0.105	0.012
African American	0.061	2533	0.140	0.163	0.392	2507	0.173	0.164	0.293
Hispanic	0.146	2100	0.078	0.144	0.590	2053	0.031	0.146	0.830
Meta-analysis			0.179	0.075	0.017		0.182	0.076	0.016

Allele frequencies and estimated effects are reported for the effect allele T (versus the reference allele C). Model 1 includes adjustment for age, sex, study site, and principal components of ancestry. Model 2 includes all covariates in Model 1, with the addition of LDL-C. Estimated effect sizes are presented based on an additive genetic model with 1 degree of freedom, for the logistic regression of CAC (presence/absence).

### *ID3* SNP rs11574 and atheroma burden in CAVA

**Figure 1:**
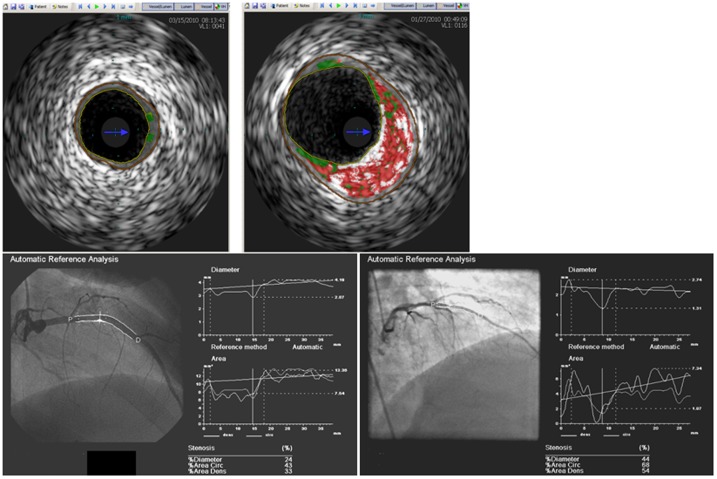
(top panel) Representative IVUS frame from a patient with the ancestral allele (left) and a patient with the risk allele (right). Light green represents fibrofatty plaque, dark green fibrous plaque, red necrotic core, and white calcium. (bottom panel) Representative QCA from a patient with the ancestral allele with a 28% stenosis (left) and a patient with the risk allele and stenosis of 73% (right). Automated edge detection is used to determine stenosis severity.

**Figure 2:**
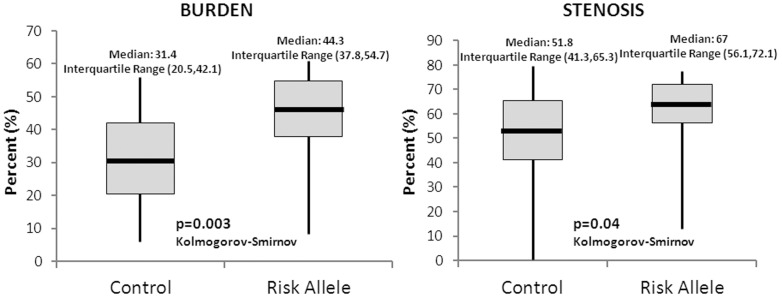
Effect of the risk allele on burden and stenosis as measured by IVUS. Burden differed significantly between groups with greater burden in the presence of the risk allele. Stenosis was marginally significant. Vertical line represents complete spread of the data. Burden represents the percent of the vessel wall occupied by plaque. Stenosis represents percent luminal stenosis.

Given that CAC is a predictor of coronary disease burden, we sought to determine directly if the *ID3* SNP rs11574 is associated with atheroma burden measured by IVUS and with stenosis severity by QCA in a second cohort. A representative IVUS and QCA from patients with and without the risk allele are shown in **Figure 1**. Subjects with the risk allele demonstrated a significantly greater atheroma burden than subjects without the risk allele (*P* = 0.003). Additionally, while QCA is a cruder measure of disease burden and has less ability to describe the full extent of disease, there was also a significant difference in stenosis between groups (*P* = 0.04) (**Figure 2**). Minimal lumen area also differed significantly between groups (Risk allele (6.13+/−3.02, Ancestral allele 4.37+/−2.07; p value 0.0158). Multivariate analysis was performed with 2 models as shown in Table 3. The risk allele remained the most predictive covariate of atheroma burden in multivariate analysis (Model 1 covariates: age, genotype, gender, and LDL, p = 0.0110, regression coefficient = 9.578, SE = 3.657; Model 2 covariates: Model 1, presence of hypertension, presence of diabetes, p = 0.0163, regression coefficient = 9.294, SE = 3.767, p = 0.0163).

**Table 3:** Multivariate predictors of Atheroma Burden and Stenosis.

Multivariate Predictors of Burden							
MODEL 1	Beta	SE	p-value	MODEL 2	Beta	SE	p-value
Age	0.325	0.175	0.0671	Age	0.325	0.177	0.0714
Genotype	9.578	3.657	0.0110	Genotype	9.294	3.767	0.0163
Gender	−1.527	3.393	0.6542	Gender	−1.276	3.479	0.7151
LDL	−0.053	0.056	0.3521	LDL	−0.046	0.059	0.4322
				Hypertension	−1.402	4.073	0.7318
				Diabetes	1.652	3.939	0.6764

Multivariate Predictors of Stenosis							
MODEL 1	Beta	SE	p-value	MODEL 2	Beta	SE	p-value
Age	0.375	0.229	0.1061	Age	0.382	0.231	0.1034
Genotype	8.389	4.788	0.0846	Genotype	8.251	4.913	0.0980
Gender	−3.007	4.442	0.5008	Gender	−3.076	4.537	0.5002
LDL	−0.071	0.074	0.3354	LDL	−0.069	0.077	0.3708
				Hypertension	4.181	5.312	0.4342
				Diabetes	5.137	0.180	0.8606

## Discussion

Atherosclerosis is a complex disease linked to both environmental and genetic risk factors. Population studies support an important role for environmental factors, most notably a Western lifestyle [16], [17]. Yet, atherosclerosis is clearly a heritable disease [18] and identification of gene variants associated with atherosclerosis holds promise to lead to novel biomarkers and new treatment strategies based on previously unknown regulatory pathways. In order to study the complexities of human coronary artery atherosclerosis and to investigate novel murine findings in the human model, one must have a reliable manner of phenotyping disease that is predictive of coronary events.

IVUS has emerged as the gold standard for measuring atheroma burden in a precise manner. IVUS has the ability to quantitate total atheroma volume in the artery wall whether or not it is visible on angiography. This allows for study of not only overt coronary artery disease but also earlier stages of disease that are otherwise not easily detectable. It is not surprising that the rs11574 SNP predicts early disease as represented by burden rather that late disease as measured by stenosis as other factor such as shear stress and endothelial damage may be at play in the presence of late, hemodynamically significant lesions. Atheroma burden by IVUS has been linked strongly to clinical events, even more so than traditional, well-accepted clinical risk variables [2], [3]. Noninvasively, both cIMT and CAC have been shown to be predictive of coronary disease events and may be population dependent in their predictive ability. CIMT appears to be most predictive in White diabetic populations while CAC is most predictive in healthier populations at moderate rather than high risk for coronary events such as that found in MESA [4]. Thus, the proper choice of surrogate endpoints may depend on the stage of disease being studied and the population in which it is being studied. Careful phenotyping can provide a model whereby associations of coronary disease with novel pathophysiologies discovered in murine models can be initially tested in humans.

Genome-wide association studies (GWAS) have allowed for analysis of millions of genotyped SNPs as well as additional imputed SNPs to identify variant gene loci associated with increased risk for coronary artery disease. However, because GWAS applies an unbiased approach to discovery of novel genomic variants, they employ very strict thresholds for statistical significance that account for multiple testing across millions of SNPs. By their nature, GWAS studies fail to take into account the wealth of existing knowledge in biological associations, from both human and animal studies, that have accumulated over the past several decades of research. Here, we utilized a more targeted approach, testing for association of one SNP in a gene that is shown to be atheroprotective in multiple mouse models of atherosclerotic disease, and associated with cIMT in a cohort of White individuals with type 2 diabetes. Previous studies using genome-wide linkage analysis in humans and an interspecific genetic cross in mice identified risk for early MI and atherosclerosis associated with loci in which ID3 resides [19], [20]. Testing only for association of the ID3 SNP rs11574, we found association of this SNP and coronary artery disease in two cohorts; CAC in MESA and IVUS coronary artery plaque volume in CAVA, a cohort of White patients undergoing cardiac catheterization.

Comparing the results across race/ethnic groups in MESA, we found the strongest association for Whites, with weaker evidence for association in African Americans and Hispanics. The direction of effect for the rs11574 SNP was observed to be consistent across all race/ethnic groups, suggesting a common functional mechanism lies behind the association in all three groups. The difference in statistical significance across MESA race/ethnic groups may be due in part to reduced frequency of the risk allele in MESA African Americans (6.1%) and Hispanics (14.6%) as compared to MESA Whites (23.9%). The attenuated effect estimates seen in African Americans and Hispanics also indicate there may be other genetic and environmental factors modifying the association between the *ID3* SNP rs11574 and CAC in MESA participants. For example, behavioral and dietary differences across race/ethnic groups may overlap with the functional pathway of the *ID3* SNP, leading to differences in SNP effects across these groups. In addition, there may be undetected epistatic interactions with SNPs in other genes across the genome. Strong differences in allele frequencies across race/ethnic groups for such SNPs could modify the observed effect for the *ID3* SNP when estimated without taking into account this type of interaction.

The finding that a SNP in a single gene could be associated with a complex polygenic disorder such as atherosclerosis is supported by the fact that Id3 regulates multiple genes within pathways and cell types linked to atherosclerosis. T cells have important roles in atherosclerosis, and Id3 has been implicated in T cell maturation, differentiation [21]–[25], and T cell driven autoimmune disease [26]–[28]. In addition to expression and function within the immune system, the broadly expressed Id3 protein has also been implicated to function in vessel wall cells. In fact, bone marrow transplant studies in mice demonstrate that Id3 mediated atheroprotection is not all due to bone marrow derived cells [29]. 12/15-lipoxygenase, an important enzyme that modifies LDL in the sub-endothelial space of the atheroma, can upregulate Id3 expression in VSMCs and subsequently proliferation of VSMCs [30], [31]. Additionally, VCAM-1, an important adhesion molecule for leukocyte recruitment to the vessel wall, is upregulated in VSMCs and whole aortas isolated from *Id3^−/−^* mice [12]. These studies suggest that Id3 has an important role in vessel wall cells to suppress pro-atherogenic processes.

The majority of published data in murine models of atherosclerosis, link Id3 to B cell-mediated atheroprotection. In mice, B cells are present in atherosclerosis prone regions of the aorta, prior to onset of disease. Loss of B cells in these regions is associated with increased atherosclerosis. Compared to control *Apoe^−/−^* mice, *Id3^−/−^Apoe^−/−^* mice had a dramatic reduction in aortic B cells and a marked increase in atherosclerosis. Additionally, adoptive transfer of *Apoe^−/−^* B cells to B cell deficient *μMT Apoe^−/−^* mice attenuated atherosclerosis, an effect that was lost when the *Apoe^−/−^* B cells were null for Id3. A role for B cells in human atherosclerosis is supported by the ability of human B cells to produce antibodies specific to modified lipids [32] and demonstrated associations between circulating antibodies to modified lipids and CAD [33], [34]. As B cell-mediated atheroprotection in mice depends on Id3, the association of the functional polymorphism in *ID3* with measures of atherosclerosis [11] in humans raises the intriguing hypothesis that ID3 may mediate B cell atheroprotection in humans and the polymorphism at rs11574 may identify those with loss of this humoral immune protection.

The current study does not address these or other mechanisms whereby polymorphism at rs11574 in the human *ID3* gene is associated with increased coronary artery plaque volume and calcium accumulation. Yet, taken together, data provide for the generation of novel hypotheses that could be tested in humans and lead to not only novel biomarkers of disease, but novel strategies for disease prevention.

## Supporting Information

File S1 **Combined supporting information file including Supplementary Methods and Table S1.** Table S1: Characteristics of MESA Whites, African Americans and MESA Hispanics by rs11574 genotype.
(DOCX)
