# Correction: A Functionally Significant Polymorphism in *ID3* Is Associated with Human Coronary Pathology

**DOI:** 10.1371/journal.pone.0109222

**Published:** 2014-09-19

**Authors:** The PLOS ONE Staff

The following information is missing from the Funding section of the published article: MESA Air is conducted and supported by the US Environmental Protection Agency in collaboration with MESA Air investigators, with support provided by grant RD83169701.
