# Supplementary material for: A Functionally Significant Polymorphism in ID3 Is Associated with Human Coronary Pathology
Source: PLoS One. 2014 Mar 6;9(3):e90222. doi: 10.1371/journal.pone.0090222 (PMC3946163; doi:10.1371/journal.pone.0090222)
Supplement: File S1 — Combined supporting information file including Supplementary Methods and Table S1. Table S1: Characteristics of MESA Whites, African Americans and MESA Hispanics by rs11574 genotype. (DOCX) [file pone.0090222.s001.docx]

# Supporting Information for “A Functionally Significant Polymorphism in *ID3* is Associated with Human Coronary Pathology”

Ani Manichaikul, Stephen S. Rich, Heather Perry, Joseph Yeboah, Michelle Law, Molly Davis, Matthew Parker, Michael Ragosta, Coleen A. McNamara, Angela M Taylor

**Supplementary Methods**

*Phenotyping of MESA participants*

The MESA cohort measures of subclinical atherosclerosis (SCA) included the presence or absence of CAC as a dichotomous phenotype, described previously [1]. Risk factors of atherosclerosis were recorded for participants. Participants were classified as diabetic using the 2003 ADA fasting criteria (fasting glucose >=126 mg/dL) [2] and/or diabetes treatment. Hypertension was defined according to the JNC VI (1997) criteria [3].

*MESA Genotyping*

Participants recruited by the MESA cohort (6,814) and two ancillary studies – MESA Family Study (MESAFS with 2,128 participants from 528 families) and MESA Air (5,479 participants from MESA, 257 external to MESA, and 490 from MESAFS) were genotyped in 2009 using the Affymetrix Human SNP array 6.0 (~1 million SNPs). Genotype quality control for these data have been described previously [4]. Briefly, data was filtered on SNP level call rate < 95%, individual level call rate < 95%, heterozygosity > 53%, and all monomorphic SNPs were removed. The cleaned genotypic data was deposited with corresponding MESA, MESAFS and MESA AIR phenotypic data into dbGaP under the MESA SHARe project (study accession phs000209) for 8,224 consenting individuals (2,685 non-Hispanic White, 777 Chinese, 2,588 non-Hispanic African-American, and 2,174 Hispanic) with 897,981 SNPs passing study specific quality control.

*Principal component analysis in MESA*

Principal component analysis (PCA) was performed to adjust for population structure among all MESA participants, as described previously [4]. Briefly, subsets of genotyped SNPs were constructed and thinned for linkage disequilibrium (LD). Using the LD-thinned subsets of SNPs, PCA was conducted as implemented in the program SMARTPCA [5,6] from the software package EIGENSTRAT to compute principal components (PCs) of ancestry for unrelated subsets of individuals, removing inferred first degree relatives from the analysis. Histograms and QQ plots were constructed to assess symmetry and normality of the distribution of loadings for each of the resulting PCs to determine the optimal number of PCs to include in genetic association analysis.

*Imputation of the ID3 SNP rs11574 in MESA*

Because the *ID3* SNP rs11574 was not directly genotyped in MESA, rs11574 genotypes were imputed using the genome-wide genotypes from the Affymetrix 6.0 GWAS array. IMPUTE version 2.1.0 was used to perform imputation for all MESA participants (chromosomes 1-22) using HapMap Phase I and II - CEU+YRI+CHB+JPT as the reference panel (release #22 - NCBI Build 36 (dbSNP b126)). (Only the CEU reference panel was used for imputation in White participants). Using the observed versus expected variance metric [7] to quantify imputation quality, a threshold was set at an imputation quality > 0.5 for inclusion in the current investigation. The ID3 SNP rs11574 was imputed with good quality in MESA Whites, African Americans, and Hispanics (imputation quality scores of 0.60, 0.67, and 0.53, respectively), but with poor imputation quality in Chinese-Americans (imputation quality score of 0.10). For subsequent analyses, Chinese Americans are excluded for analysis of rs11574.

*Genetic association analysis in MESA*

Genetic association analysis was performed in Whites, African Americans, and Hispanics. Individuals from the full MESA cohort (including participants from MESA, MESA FS, and MESA Air) were used to select participants for the analysis. Participants were stratified by racial/ethnic group, and those individuals with top PCs of ancestry > 3.5 SD from the mean within any racial/ethnic group were excluded. To allow study site to be included as a covariate in genetic association analysis within each racial/ethnic group, the data set was restricted to individuals from study sites with data available for at least 20 individuals of that racial/ethnic group. For each of the phenotypic analyses, the data set was restricted to individuals with data available for the particular phenotype of interest. For quantitative traits, outliers were defined as individuals with phenotypic values more than 3.5 SD from the mean, with the mean and SD calculated separately for each of the stratified analyses performed. Analyses of cIMT and iIMT as continuous phenotypes were performed on the log-scale, yielding approximately normal phenotypic distributions. Analyses of CAC (presence / absence) as a dichotomous phenotype were performed by logistic regression.

Initially, stratified analyses were performed within each racial/ethnic group. For analysis of Whites, an unrelated subset of individuals was first constructed by selecting at most one individual from each pedigree based on inferred relationships in KING [8], linear regression of quantitative phenotypes or logistic regression of dichotomous phenotypes was performed in R [9]. For analysis of phenotypes with a substantial familial component among African American and Hispanic cohorts, analysis was performed using an additive model with a linear mixed-effects model for quantitative traits, or generalized estimating equations for dichotomous traits, to account for familial relationships as implemented in the package R/GWAF [10].

All analyses were initiated with a basic model including age, sex, study site and PCs of ancestry. (Based on our examination of PCs within each racial/ethnic group, as described above, 3 PCs were used for analysis of Whites, 1 PC for African Americans, and 3 PCs for Hispanic Americans). To examine sensitivity of our results to levels of LDL cholesterol, genetic association analysis was performed under a second model that added LDL to the basic model.

Following stratified analyses, meta-analysis was performed to combine results across all four racial/ethnic groups. We performed fixed effect meta-analysis to combine estimated effects and standard errors from stratified analyses, as implemented in METAL [11].

*Intravascular Ultrasound in CAVA*

IVUS was performed on the least angulated vessel providing the longest length for evaluation in accordance with the standards of the American College of Cardiology for image acquisition on a non-infarct related artery [12]. 50U/kg of intravenous heparin and 150 mcg of intracoronary nitroglycerin were given prior to advancement of a 2.6F, 30 MHz IVUS catheter (Volcano Corporation) over a guidewire into the target vessel. The transducer was positioned distally and a motorized pullback was performed at 0.5 mm/s with images being obtained at 30 frames/second for a minimum of 30mm. The R-100 pullback device (Volcano Corporation) is a non-disposable reusable pullback device specifically designed for use in clinical trials. Each device is individually calibrated and inspected to meet the required accuracy of ± 0.03mm for pullback speeds of 0.5mm/sec. Digital images were stored for analysis off-line. Every 60^th^ image was analyzed generating cross sections spaced 1.0mm apart. Images were analyzed using VIAS (Volcano Image Analysis Software).

Atheroma burden was measured for the 40mm segment. For segments that are prohibitive to analysis secondary to calcium, the closest usable segment was substituted.

Atheroma Burden: Σ atheroma _CSA_

------------------------ x100

Σ EEM _CSA_

(EEM= external elastic membrane)

Image analysis was performed by two blinded investigators. Interobserver and intraobserver correlation coefficients for our laboratory were 0.96 and 0.98, respectively.

*CAVA Genotyping*

The sequences of all the PCR primers for *ID3* SNP rs11574 were obtained using the PyroMark design software and the genome website: www.genome.ucsc.edu. The specificity of the primers for the rs11574 site was evaluated using both bisearch (htpp://bisearch.enzim.hu/) and BLAT. The reverse primer was biotinylated. The sequences of the forward, reverse and sequencing primers were; Fw: 5’-ATTGCCTCGCGTAACTCTTCC-3’, Rev: Biotin-5’-CCGAGTCAGTGGCAAAAGC-3’ and Seq: 5’- CTAATCAGACAGCCGAG-3’ respectively. PCR reactions were performed in a final volume of 25ul containing 12.5ul of 2X Pyro Master mix, 0.5ul of 10uM forward primer, 0.5ul of 10uM reverse primer, 2.5ul of Coral load, 8ul H2O and 1ul DNA (10ng/ul). The Pryo Master mix and the Coral load were from the PyroMark Kit (Qiagen #978703). Thermal cycling was performed in a BioRad C1000 thermal cycler as follows: 95^o^C x 15 minutes, 94^o^C x 30 seconds, 60^o^C x 30 seconds, 72 ^o^C x30 seconds X 50 cycles with a final extension of 72^o^C x 10 minutes. The biotinylated PCR product was immobilized onto streptavidin-coated beads (GE-Healthcare #17-5113-01) in a binding reaction master mix consisting of 40ul binding buffer, 2ul beads, 28ul of H_2_O and 10ul of PCR reaction product in 0.2 ml tubes. An annealing reaction mix was then prepared using .3uM sequencing primer in 25ul per well of a PyroMark sequencing plate (Qiagen #979201). Annealing of the sequencing primer to the biotinylated DNA strand was performed following PyroMark Q24 protocol. Upon completion of the annealing reaction the sequencing plate was loaded into the Pryosequencer (Qiagen PyroMark Q24) and genotyping for the ID3 rs11574A/G SNP done as described previously [13].

*CAVA Statistics*

All statistics were performed using SAS 9.3. Baseline characteristics are reported as median and interquartile range. All characteristics were not normally distributed with the exception of age and BMI. Categorical data was compared using a chi square test or Fishers exact where appropriate. Continuous data was compared using the student t-test or Wilcoxon sign-ranked test where appropriate. Differences in burden and stenosis between groups were determined using a nonparametric test (Kolmogorov Smirnov) due to data distribution. Multivariate analysis was performed using a general linear regression model and Type III sum of squares.

**Table S1:** Characteristics of MESA Whites, African Americans and MESA Hispanics by rs11574 genotype.

|  | **MESA Whites** | | **MESA African Americans** | | **MESA Hispanics** | |
| --- | --- | --- | --- | --- | --- | --- |
|  | **Wildtype** | **Risk allele carriers** | **Wildtype** | **Risk allele carriers** | **Wildtype** | **Risk allele carriers** |
| **Participant characteristics*** |  |  |  |  |  |  |
| No. subjects | 1517 | 1071 | 2287 | 273 | 1650 | 480 |
| Women | 770 (50.8) | 566 (52.8) | 1298 (56.8) | 140 (51.3) | 896 (54.3) | 255 (53.1) |
| Age, years | 63.0 [54.0, 71.0] | 63.0 [54.0, 71.0] | 60.0 [53.0, 68.0] | 62.0 [55.0, 69.0] | 60.0 [53.0, 68.0] | 59.0 [52.0, 67.0] |
| BMI, kg/m^2^ | 27.2 [24.3, 30.6] | 26.9 [24.1, 30.3] | 29.4 [26.1, 33.7] | 29.6 [26.3, 34.3] | 28.6 [25.9, 32.2] | 28.6 [26.2, 31.9] |
| LDL-C, mg/dL | 115.0 [95.0, 135.0] | 116.0 [96.0, 137.0] | 116.0 [95.0, 138.0] | 113.0 [92.0, 133.8] | 118.0 [95.8, 139.0] | 117.0 [97.0, 140.0] |
| HDL-C, mg/dL | 49.0 [40.0, 61.0] | 50.0 [42.0, 61.0] | 51.0 [42.0, 61.0] | 49.0 [41.0, 62.0] | 46.0 [39.0, 55.0] | 46.0 [39.0, 55.0] |
| Triglycerides, mg/dL | 114.0 [77.0, 163.0] | 112.0 [76.0, 163.0] | 88.0 [65.0, 122.0] | 89.0 [64.8, 123.2] | 134.0 [95.0, 194.0] | 142.0 [99.0, 196.5] |
| Diabetes (yes/no) | 105 (6.9) | 59 (5.5) | 398 (17.5) | 45 (16.6) | 307 (18.6) | 89 (18.6) |
| Hypertension (yes/no) | 582 (38.4) | 412 (38.5) | 1371 (59.9) | 164 (60.1) | 693 (42.0) | 196 (40.8) |
| Lipid medication (yes/no)† | 305 (20.1) | 184 (17.2) | 423 (18.5) | 59 (21.6) | 279 (16.9) | 78 (16.2) |
|  |  |  |  |  |  |  |
| **Subclinical atherosclerosis** |  |  |  |  |  |  |
| CAC (presence / absence) | 863 (56.9) | 631 (58.9) | 971 (42.8) | 133 (49.4) | 757 (46.1) | 220 (46.3) |
| Common carotid IMT, mm | 0.84 [0.73, 0.97] | 0.85 [0.73, 0.98] | 0.86 [0.75, 0.98] | 0.89 [0.76, 1.01] | 0.81 [0.71, 0.94] | 0.81 [0.71, 0.92] |
| Internal carotid IMT, mm | 0.88 [0.71, 1.34] | 0.91 [0.72, 1.44] | 0.90 [0.70, 1.30] | 0.92 [0.71, 1.39] | 0.85 [0.69, 1.22] | 0.78 [0.66, 1.14] |

Data are presented as n (%) for binary measures or median [IQR] for continuous measure. “Wildtype” and “Risk allele carriers” refer to rs11574 genotypes. "Wildtype" denotes homozygote for the common allele (CC) and risk allele carrier indicates het (CT) or homozygote for the less frequent allele (TT). For MESA, rs11574 was imputed, so we use genotype dosage (i.e. the estimated number of copies of the risk allele) to classify individuals as wildtype (dosage in [0, 0.5) ) vs. risk allele carriers (dosage in [0.5, 2]).

*Summary statistics are reported for the subset of individuals with data available for at least one of the subclinical atherosclerosis phenotypes.

†In MESA, data are available for “any lipid lowering medication”. For IVUS, these data represent statin use.

**References**

1. Folsom AR, Kronmal RA, Detrano RC, O'Leary DH, Bild DE, et al. (2008) Coronary artery calcification compared with carotid intima-media thickness in the prediction of cardiovascular disease incidence: the Multi-Ethnic Study of Atherosclerosis (MESA). Arch Intern Med 168: 1333-1339.

2. Genuth S, Alberti KG, Bennett P, Buse J, Defronzo R, et al. (2003) Follow-up report on the diagnosis of diabetes mellitus. Diabetes Care 26: 3160-3167.

3. (1997) The sixth report of the Joint National Committee on prevention, detection, evaluation, and treatment of high blood pressure. Arch Intern Med 157: 2413-2446.

4. Manichaikul A, Naj AC, Herrington D, Post W, Rich SS, et al. (2012) Association of SCARB1 Variants With Subclinical Atherosclerosis and Incident Cardiovascular Disease: The Multi-Ethnic Study of Atherosclerosis. Arterioscler Thromb Vasc Biol.

5. Patterson N, Price AL, Reich D (2006) Population structure and eigenanalysis. PLoS Genet 2: e190.

6. Price AL, Patterson NJ, Plenge RM, Weinblatt ME, Shadick NA, et al. (2006) Principal components analysis corrects for stratification in genome-wide association studies. Nat Genet 38: 904-909.

7. Li Y, Willer CJ, Ding J, Scheet P, Abecasis GR (2010) MaCH: using sequence and genotype data to estimate haplotypes and unobserved genotypes. Genet Epidemiol 34: 816-834.

8. Manichaikul A, Mychaleckyj JC, Rich SS, Daly K, Sale M, et al. (2010) Robust relationship inference in genome-wide association studies. Bioinformatics 26: 2867-2873.

9. R Development Core Team (2010) R: A Language and Environment for Statistical Computing. R Foundation for Statistical Computing.

10. Chen MH, Yang Q (2010) GWAF: an R package for genome-wide association analyses with family data. Bioinformatics 26: 580-581.

11. Willer CJ, Li Y, Abecasis GR (2010) METAL: fast and efficient meta-analysis of genomewide association scans. Bioinformatics 26: 2190-2191.

12. Mintz GS, Nissen SE, Anderson WD, Bailey SR, Erbel R, et al. (2001) American College of Cardiology Clinical Expert Consensus Document on Standards for Acquisition, Measurement and Reporting of Intravascular Ultrasound Studies (IVUS). A report of the American College of Cardiology Task Force on Clinical Expert Consensus Documents. J Am Coll Cardiol 37: 1478-1492.

13. Nordfors L, Jansson M, Sandberg G, Lavebratt C, Sengul S, et al. (2002) Large-scale genotyping of single nucleotide polymorphisms by Pyrosequencingtrade mark and validation against the 5'nuclease (Taqman((R))) assay. Hum Mutat 19: 395-401.
